# Differential profiles of reading development in neurodevelopmental disorders under an adapted tier 2 response to Intervention protocol

**DOI:** 10.3389/fped.2026.1737520

**Published:** 2026-04-20

**Authors:** Renata Mousinho, Fernanda Mesquita, Lia Pinheiro, Cristina Leitão, Rosinda Martins Oliveira, Felipe Pegado, Erika Carvalho Rodrigues

**Affiliations:** 1 Speech Therapy Department, School of Medicine, Federal University of Rio de Janeiro, Rio de Janeiro, Brazil; 2 Psychology Institute, Federal University of Rio de Janeiro, Rio de Janeiro, Brazil; 3 Laboratory for the Psychology of Child Development and Education (LaPsyDÉ), Université Paris Cité, Paris, France; 4 D'Or Institute for Research and Education, IDOR, Rio de Janeiro, Brazil

**Keywords:** attention-deficit/hyperactivity disorder, borderline intellectual functioning, dyslexia, reading comprehension, reading speed, response to intervention

## Abstract

**Introduction:**

Reading proficiency is a foundational skill. Failure to achieve reading competence constitutes a global educational and public health challenge. This burden is especially high among children with neurodevelopmental conditions. Despite the availability of evidence-based frameworks, such as Response to Intervention (RTI), a lack of scalable, context-sensitive models remains for supporting struggling readers in low-resource environments. Building on this context, this study describes the Less Intensive Response to Intervention Tier 2 (LIRTI^2^). We hypothesize that the LIRTI^2^ will improve reading speed and comprehension in children with Attention-Deficit/Hyperactivity Disorder (ADHD), Borderline Intellectual Functioning (BIF), and those at risk for Dyslexia (arDYS). We also predict differential effectiveness of the intervention across neurodevelopmental profiles.

**Methods:**

This retrospective, service-based study included 90 children (median age = 9 years, 3rd grade). Participants (ADHD = 37, BIF = 14, and arDYS = 39) completed 18 weekly, two-hour sessions per week that combined explicit phonological awareness and fluency-focused instruction with playful, low-cost materials. For the intervention, students were divided into small groups based on reading proficiency. Reading speed (words per minute) and reading comprehension (literal questions) were assessed before and after the intervention. The groups were similar in age, school grade, and sex distribution.

**Results:**

A significant diagnostic effect was found on post-intervention reading speed, after adjusting for baseline reading speed and schooling [ANCOVA F(2,85) = 4.345, *p* > 0.01]. The ADHD group demonstrated significantly higher reading speed than BIF (*p* = 0.034) and arDYS (*p* = 0.047), whereas the BIF–arDYS comparison was not significant. While clinical group was not associated with reading comprehension level (low, medium, high) before LIRTI^2^, this association became significant after the intervention [*χ*^2^(4, *N* = 90) = 14.75, *p* = 0.005]. Adjusted standardized residuals indicated that more children with ADHD achieved “high” comprehension levels.

**Discussion:**

LIRTI^2^ is an out-of-school, small-group intervention with potential scalability in low-resource settings where access to services is limited. Reading fluency and comprehension improved following the intervention, with larger gains in children with ADHD than in those with BIF or arDYS. Future studies with follow-up are needed to confirm which learner profiles benefit most and to determine the intervention’s broader academic impact.

## 1 Introduction

Reading is a critical prerequisite for full participation in democratic societies and the exercise of citizenship (1). Functional reading goes beyond basic decoding, encompassing the capacity to understand, interpret, and apply written information in diverse settings, including societal and institutional domains (2). Despite global advances, over 770 million adults remain illiterate (1), and many others lack functional reading, even in high-income countries, due to educational inequities (3). In Brazil, only 12% of adults demonstrate full functional reading (4), and students score significantly below the OECD (Organisation for Economic Co-operation and Development) Programme for International Student Assessment (PISA) reading average (5).

Reading fluency can be defined as the ability to accurately recognize and decode words, read at an appropriate pace (reading speed), and employ natural phrasing, intonation, and rhythm (expressivity) to effectively convey meaning and support reading comprehension (6). Reading acquisition is shaped by a dynamic interplay of neurobiological and environmental factors (7).

It is important to note that an increase in reading speed throughout formal schooling is well documented internationally. As children gain continued exposure to written language alongside systematic instruction and practice, their reading becomes progressively more fluent and automatic (8, 9). Studies conducted across diverse linguistic and educational contexts consistently demonstrate that reading speed develops progressively from the early primary years through later schooling—a trajectory shaped by cognitive maturation, instructional quality, and increasing familiarity with written language (10, 11). In Brazil, similar findings have been reported, highlighting the progressive development of reading fluency across grade levels. The literature emphasizes the critical role of schooling in fostering gradual improvements in reading speed, regardless of socioeconomic and instructional variability (12–14).

Difficulties in the reading acquisition process, irrespective of their origin, are frequently associated with significant consequences. Among children with neurodevelopmental disorders, as classified in the Diagnostic and Statistical Manual of Mental Disorders, Fifth Edition, Text Revision (DSM-5-TR), including Dyslexia, Attention-Deficit/Hyperactivity Disorder (ADHD), and borderline intellectual functioning (BIF), reading impairments may lead to persistent academic and socio-emotional consequences (15).

Dyslexia is classified as a Specific Learning Disorder with impairment in reading (DSM-5-TR), characterized by difficulties in reading fluency and orthographic processing that are disproportionate to the individual's development in other cognitive or academic domains (16, 17). In contrast, ADHD is characterized by persistent patterns of inattention, hyperactivity, and impulsivity that cause significant functional impairments (18), which can hinder the development of reading fluency (19, 20). Meanwhile, children with BIF are characterized by intellectual abilities that are below average but not low enough to meet the criteria for Intellectual Disability (21). Those children tend to show global developmental delays, including difficulties in reading acquisition, consistent with broader impairments across cognitive and adaptive domains (22).

Over the past decades, numerous intervention programs have been developed and tested to support at-risk readers, with a growing body of evidence highlighting the effectiveness of explicit instruction in foundational reading skills. These foundational skills typically include systematic teaching of phonemic awareness, phonic coding, and spelling, as well as the development of reading fluency (23, 24). However, successful remediation often requires a comprehensive approach that integrates vocabulary enhancement, reading comprehension strategies, and motivational support to address the multifaceted nature of reading difficulties (25, 26). One framework that encompasses these essential elements within a data-driven, multi-tiered system is Response to Intervention (RTI). The layered RTI model (27) consists of additive instructional components across three tiers: all students receive instruction in Tier 1, which includes universal screening conducted in the classroom; students demonstrating difficulties in Tier 1 participate in small-group interventions in Tier 2; and finally, students with persistent difficulties after Tiers 1 and 2 receive individualized intensive interventions in Tier 3, indicating the presence of a learning disorder.

In numerous countries, particularly within the United States (28), the Response to Intervention model has been implemented as a proactive approach to address developmental challenges in educational settings. This structured, multi-tiered framework facilitates early identification and support for children requiring additional assistance (29, 30). Recent international studies also highlight the effectiveness of programs for reading interventions implemented outside formal school environments, particularly within community-based or extracurricular settings. These interventions are typically designed for students identified as being at risk for reading difficulties—those who do not make adequate progress following high-quality core instruction (like Tier 1), yet do not require the more intensive, individualized, specialized support characteristic of Tier 3 interventions (31, 32). Evidence from initiatives in countries such as Ghana, Ethiopia, South Africa, the United Kingdom, and Australia indicates that structured reading clubs, and targeted, skill-specific instructional models can lead to measurable improvements in key foundational reading skills, including decoding, fluency, and comprehension (33–36).

Despite its international adoption, the implementation of two-tier RTI practices related to reading development in Brazil remains very limited. Existing efforts remain scarce and are generally restricted to isolated initiatives with small sample sizes, primarily aimed at exploratory investigation (37–45). Although Brazilian education policy has expanded legal guarantees for children with disabilities, conditions such as Dyslexia and Attention Deficit Hyperactivity Disorder (ADHD) are not yet fully included within the scope of inclusive education. However, a law seeks to provide comprehensive support for students with Dyslexia, ADHD, and other learning disorders, with particular emphasis on identification, without offering alternatives to address learning difficulties prior to referral for formal diagnosis (46–48).

The Writing, Reading, and Orality program (ELO, from the Portuguese Escrita, Leitura e Oralidade) is a project implemented by the Federal University of Rio de Janeiro that specializes in supporting children with reading disorders. It integrates interdisciplinary diagnostic assessments and implements adapted Tier 2 and Tier 3 interventions within the RTI (Response to Intervention) framework. The adapted Tier 2, here referred to as LIRTI^2^ (Less Intensive Response to Intervention—Tier 2) is specifically designed for implementation beyond the school environment, as demonstrated in other studies (49). The LIRTI^2^ is delivered over 18 weeks, in weekly 2 h sessions, to small groups (3–5 students). Students are grouped based on reading proficiency for optimizing instructional effectiveness and addressing individual learning needs (50). Furthermore, arranging by reading level fosters a positive learning environment where students are more likely to engage collaboratively and feel motivated, thereby reducing frustration among struggling learners and boredom among more advanced peers (51).

Recently, researchers (52) found that students who demonstrate stronger reading fluency during the first two years of formal education tend to achieve greater gains in reading performance throughout the remainder of primary school. These findings reinforce the critical value of early and targeted interventions, such as those provided through Tier 2 of the Response to Intervention (RTI) model.

Building on this context, this study describes LIRTI^2^'s simplified structure and delivery in under-resourced, school-adjacent settings and estimate treatment response in reading speed and reading comprehension, examining whether outcomes differ across diagnostic subgroups (ADHD, BIF and at risk for Dyslexia—arDYS). Reading speed was selected as the primary outcome, measured in words per minute in oral text, because it integrates decoding and fluency, serving as a proxy for reading competence and a sensitive marker of reading disorders (53). Reading comprehension was assessed with literal questions on grade-appropriate narrative texts (52).

By analyzing routinely collected service data, we address a critical evidence gap on Tier 2 supports for at-risk readers in Brazil.

Based on previous evidence, we hypothesize that the LIRTI^2^ will be effective in improving reading skills in children with neurodevelopmental disorders, including ADHD, BIF and arDYS. Specifically:

1. Children receiving the LIRTI^2^ intervention will show improvements in reading speed.
2. The intervention will lead to measurable gains in reading comprehension.
3. The effectiveness of the intervention will differ across neurodevelopmental profiles.

## 2 Methods

### 2.1 General ELO program description

ELO is a program that aims to support children and families experiencing reading difficulties at the time at the Deolindo Couto Institute of Neurology (CEP/INDC), approval number 09/2010), Federal University of Rio de Janeiro, Brazil. Referrals for the program originated from school teachers who identified students with learning challenges, medical professionals, or self-referrals from families prompted by information disseminated through multiple sources, including community organizations and media.

Within the ELO initiative, the intake visit involved two simultaneous components: a structured caregiver interview to obtain medical, developmental, and educational history, and a brief developmental screening with the child. If this evaluation indicated that the case was indicate for the program, the child would advance to a comprehensive interdisciplinary assessment.

Interdisciplinary assessment sessions conducted by professionals in neurology, neuropsychology, speech therapy, audiology, and psychopedagogy were typically carried out over two days. Each assessment lasted approximately one hour, with scheduled breaks between sessions to avoid fatigue. To prevent overloading the child, no more than three assessments were conducted per day. Following completion of the evaluations, all results were reviewed in an interprofessional case conference attended by the involved professionals, where a preliminary diagnosis and recommendations were established.

### 2.2 Participants: from project ELO to LIRTI^2^

ELO Participants with reading difficulties and aged between 6 and 14 years old were invited to LIRTI^2^ small group reading intervention. Exclusion criteria to the LIRTI^2^ included the presence of severe oral language disorders. The ELO Project is a reference center for Specific Learning Disorders. Accordingly, children with severe oral language disorders (being non-verbal, presence of intellectual disability, presence of agrammatism, unintelligible speech, significant impairment in comprehension), after the initial evaluation process carried out by the multidisciplinary team (speech-language pathology, psychology, neurology, pedagogy), were referred to another health service that could provide appropriate care for this type of demand. In this study, individuals with comorbid neurodevelopmental disorders were also excluded from this analytic cohort.

In the 6 years period analyzed in the present study (2010 to 2016), 524 children were enrolled in the ELO program. Of these, 155 were referred to the LIRTI^2^ small-group intervention and agreed to attend the weekly sessions; all met the predefined adherence threshold (≥90% of scheduled sessions).

**Figure 1:**
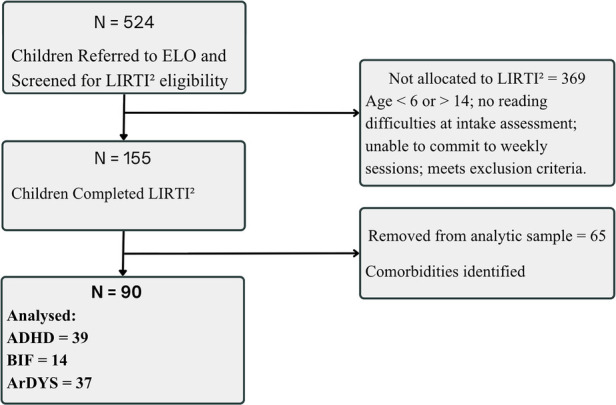
Flow diagram.

Among them, 90 were classified according to DSM-IV or DSM-5 criteria as having Attention-Deficit/Hyperactivity Disorder (ADHD); Borderline Intellectual Functioning (BIF) or at risk for Dyslexia (arDYS), as described below. Figure 1 shows the ﬂow diagram of the study.

- Attention-Deficit/Hyperactivity Disorder (ADHD): Characterized by persistent symptoms of inattention, hyperactivity, and impulsivity, significantly interfering with academic performance.
- Borderline Intellectual Functioning (BIF): Classified under “Other Conditions That May Be a Focus of Clinical Attention” in the DSM-5. These individuals presented IQ (Intelligence Quotient) scores one to two standard deviations below the mean, often accompanied by deficits in academic achievement and cognitive functioning.
- At risk for Dyslexia (arDYS): Composed of children showing a Dyslexia-consistent profile with Specific Learning Disorder with impairment in reading, particularly affecting accuracy and fluency. This label is applied descriptively and does not indicate confirmed clinical diagnoses at the time, but rather reflects a risk profile aligned with current educational and developmental screening practices, as outlined in the DSM-5.

Although the children were already followed by the service and presented a preliminary clinical profile, the intervention was intentionally framed as Tier 2 rather than Tier 3. At the time of entry into the program, a formal diagnosis had not yet been established according to DSM-5 criteria, and the initial assessment alone was not sufficient to confirm the diagnosis. Within this model, Tier 2 is defined as a targeted, time-limited intervention aimed at children at risk, characterized by moderate intensity, partial individualization, and systematic progress monitoring, but not by fully individualized, diagnosis-driven treatment. The primary goals at this tier are to provide structured support, observe the child's response to intervention, and refine clinical hypotheses. Fidelity and monitoring were ensured through standardized procedures and regular team supervision. Only after the implementation of Tier 2 intervention and the analysis of the children's response patterns was it possible to confirm the diagnosis and, when indicated, transition to Tier 3, which corresponds to highly individualized, intensive interventions explicitly guided by an established diagnosis.

### 2.3 LIRTI^2^ proposal

The Less Intensive Response to Intervention Tier 2 program is a small-group, out-of-school intervention targeting fluency, and comprehension (Table 1). LIRTI^2^ one 2-hour session per week over 18 weeks (36 h across one academic semester) in groups of 3–5 students. Pre- and post-testing comprised oral reading speed (words per minute, WPM) and literal-level comprehension. This approach was not as intensive as those described in the aforementioned studies, primarily due to financial, transportation, and work-related constraints faced by participating families, as well as a shortage of specialized personnel.

**Table 1:** LIRTI^2^ general proposal.

General Framework of the LIRTi^2^ Intervention		
Domain	Description	
Primary Objective	Improvement of reading speed	
Secondary Objective	Enhancement of reading comprehension	
Pre-intervention Assessment	Measurement of oral reading speed and comprehension	
Intervention Intensity	One session per week	
Session Duration	2 h per session	
Total Duration	18 sessions (36 h total) over one academic semester	
Group Size	3–5 students	
Instructional Approach	Play-based instructional strategies	
Home Component	Parental/caregiver involvement to support reading strategies	
Post-intervention Assessment	Measurement of oral reading speed and comprehension	
Specific Characteristics by Reader Profile		
Component	Emergent Readers	Low-Fluency Readers
Instructional Focus	Syllabic awareness; phonemic awareness; letter-sound correspondence; alphabetic principle	Fluency training; phonological awareness; vocabulary expansion; narrative skills (comprehension and production)
Materials	Play bricks; modeling clay; object images; mirrors; mouth-shape cards; vocabulary words banks; Image supports for story context	Estrelinha Collection; poster boards; markers; word cards; collective games; RAN boards

LIRTI^2^, Less Intensive Response to Intervention Tier 2.

Throughout the entire intervention process low-cost materials were used (e.g., manipulatives, image supports, word banks, books, classroom-style boards, and pencils) and a play-based approach was prioritized. This approach not only supported attention and enjoyment, but also contributed to the consolidation of learning, by reducing cognitive overload and promoting a positive emotional climate. Additionally, parents and caregivers were actively encouraged to reinforce activities at home, fostering continuity and supporting skill generalization beyond the weekly LIRTI^2^ small group sessions.

#### 2.3.1 Evaluations

Oral text reading speed and comprehension were evaluated, both pre- and post- LIRT^2^ intervention. Assessments were conducted using narrative texts appropriate for the children's grade level (12). Reading speed was measured in words per minute (WPM). Oral reading comprehension was assessed by asking four (4° and 7° grades) to five (2°, 3° 5° and 6° grades) questions about literal information from the text. The materials are not derived from a nationally validated instrument; rather, they consist of grade-appropriate texts that were previously used in a longitudinal study conducted in Rio de Janeiro, the same context in which the present study was developed, given the limited availability of validated instruments for these specific purposes at the time. The variation in the number of questions across texts reflects the attempt to ensure full coverage of each narrative through literal comprehension questions, resulting in four questions for some texts and five for others. This approach supports the comparability between the two conditions. To address this issue, reading comprehension performance was distributed based on the percentage of correct responses. Comprehension outcomes were classified as low (0%–25% correct), medium (26%–74% correct), or high (75%–100% correct). The number of participants within each diagnostic group (ADHD, BIF, and arDYS) was then categorized according to these comprehension levels.

#### 2.3.2 Interventions

LIRTI^2^ was collaboratively designed by professionals in speech-language pathology and pedagogy, focusing on enhancing reading skills. Children with profiles of ADHD, BIF, and arDYS were assigned to one of two different types of intervention levels (emergent readers or low-fluency readers). The intervention included 26 beginner readers (ADHD = 5, FIB = 7; arDYS = 14) and 64 low-fluency readers (ADHD = 34, FIB = 34; arDYS = 23).

Participants were classified as emergent readers when their reading speed was below 30 words correctly per minute (WCPM). This threshold was selected based on established international benchmarks of oral reading fluency. Specifically, normative and longitudinal studies indicate that reading speed at this level corresponds to the extreme lower end of the performance distribution, even in the early years of elementary school, and are associated with persistent deficits in decoding and reduced automaticity (54, 55). To date, no quantitative benchmarks for reading speed have been established by the Brazilian Ministry of Education, whose policy documents address reading fluency only at a qualitative level. However, minimum cut-off values for reading speed and accuracy required to support text comprehension in the second grade have been empirically estimated at 47 WCPM in Brazilian samples (56), and studies published in speech-language pathology and education journals have reported fluency levels of similar magnitude among children with reading difficulties in the early school years (57). Accordingly, a cutoff of 30 WCPM represents a conservative criterion, consistent with an early developmental stage characterized by limited automaticity in word recognition and insufficient fluency to support efficient text comprehension (58). In clinical and educational contexts, performance at this level is typically interpreted as indicative of significant reading difficulty.

Low-fluency readers, on the other hand, have begun the formal reading process, reading more than 30 WPM, but continue to experience difficulties with reading fluency. Independent of the group, LIRTI^2^ encompassed foundational reading predictors (phonological awareness, alphabet knowledge, oral language) identified by the National Early Reading Panel (24), and evidence-based strategies from Brazilian studies (37, 59, 60). The intervention sought to improve reading speed as a pathway to comprehension, since faster reading can enhance understanding by reducing the cognitive load involved in decoding (61, 62).

##### 2.3.2.1 LIRTI^2^ for emergent readers

For the emergent readers group, the intervention followed a progressive, explicit sequence of metaphonological instruction moving from syllable-level awareness toward letter–sound integration. Early sessions introduced syllabic segmentation and blending with concrete supports and picture cues; instruction then addressed initial-syllable identification and alliteration, rhyme detection, and broadened focus to medial and final syllables, integrating storytelling. Sentence repetition, comprehension questions, and retellings were practiced to booster phonological working memory.

Subsequent sessions prioritized syllable manipulation and generalization. Pattern-based reading at the syllable level and the development of a personal syllabary was stimulated. Alphabet knowledge was reinforced via letter–sound mapping and articulatory explanations. The sequence was concluded with transitioned pattern-based reading at the phoneme level, promoting recognition and recombination of sound–letter correspondences, and transfer of familiar phonological/orthographic patterns to improve decoding, as summarized in Table 2.

**Table 2:** LIRTI^2^ for emergent readers.

Structure of the Intervention per Session			
Days	Main Skill	Specific Objectives	Strategies
1–2	Syllabic Segmentation and Synthesis	Metaphonology Introduction of the concept of segmenting and blending syllables	Use of concrete supports (Lego, modeling clay), object images to associate sound with visual representation
3–4	Initial Syllable Identification & Alliteration	Metaphonology Discrimination of similar and different initial syllables in words	Use of mirrors, mouth shape cards, articulatory explanations.
5	Consolidation of Previous Skills	Reinforcement and integration of content from sessions 2 to 5	Cumulative tasks with playful engagement
6–7	Rhyme Identification	Metaphonology Recognition of similarities in final sound segments of words	Explicit instruction on stressed vowel, games with/without written cues, songs and short texts with rhymes
8–9	Medial and Final Syllable Identification	Metaphonology Expansion of phonological attention to different syllabic positions	Storytelling, sentence repetition, comprehension questions, retelling to strengthen phonological working memory
10	Cumulative Review	Consolidation of all previously acquired skills	Integrated tasks with playful and motivational elements
11	Syllable Manipulation	Metaphonology Development of syllabic awareness: structure and manipulation	Visual cues, use of blocks (highlighted syllables), step-by-step addition/ subtraction (final, initial, medial positions)
12–13	Syllable Transposition	Metaphonology Understanding how changing syllable order alters word meaning	Story introduction, mouth shape cards, image support, word bank with all target words accessible
14	Pattern-based reading at the syllable Level	Development awareness of the generative function of syllable recombination. Generalizing syllabic patterns to encode novel words	Promotion of a personal syllabary, developed from the segmentation of words of the logographic lexicon and the recombination of syllables to create new words.
15–16	Alphabet knowledge	Recognize and name letters of the alphabet and associate with their corresponding sounds. Understand the role of letters in word formation.	Introduction to the alphabet through letter–sound mapping, mouth shape cards, and articulatory explanations.
17/18	Pattern-based reading at the phoneme Level	Recognition of recurring sound-letter correspondences Generalizing syllabic patterns to encode novel words Enhancement of decoding skills	Recognition and recombining sound–letter correspondences. Guided generalization of familiar phonological and orthographic patterns.

LIRTI^2^, Less Intensive Response to Intervention Tier 2.

##### 2.3.2.2 LIRTI^2^ for low-fluency readers

All sessions for this group were based on the Estrelinha Collection, Level 1 (63–66), which consists of six volumes, used as instructional materials to develop automatic and meaningful reading (i.e., reading fluency with comprehension). Intended for children at the early stages of reading development, the Estrelinha Collection features mostly regular words, short texts, and a direct, repetitive language style, supported by simple and colorful illustrations. The first books introduce regular words with simple syllables to support early reading development. The final books gradually introduce more basic positional rules and more complex syllabic structures, such as three-element syllables.

Each book was explored over three consecutive sessions, aiming to provide a stable input, through repetition, and gradually increase cognitive demands to promote mastery of the orthographic system. Each set of three sessions followed a consistent structure, with only the target vocabulary adapted to the specific book being worked on. Thus, the activities in the first session of one book were mirrored in the first session of subsequent books, with only vocabulary changes. The same was applied to the second and third sessions. As previously noted, the use of playful and engaging activities was a consistent feature of the intervention. Poster boards and markers were used to create a large-format adapted board game designed for group play, incorporating key vocabulary and tasks targeting. Table 3 summaries LIRTI^2^ for low-fluency readers group.

**Table 3:** LIRTI^2^ for Low-fluency readers.

Structure of the Intervention Sessions		
Main Skill	Specific Objectives	Strategies
*High-frequency words from each book were previously selected as the basis for the following activities		
Motivation strategies	Task engagement Generating hypotheses about the text Vocabulary development Reading Comprehension	Based on the cover and adult prompts, nomination of illustrated elements and formulation of hypotheses about the story's content.
Identification of Grapheme/Phoneme relationship	Visual input Reading accuracy Reading expressivity	Shared reading: a collaborative reading practice in which the adult, pointing the correspondent letter, and children, read a text together. It is important to note that, even while maintaining a slower reading pace, extended pauses were avoided in order to promote a more fluid reading.
Narrative skills	Improvement of Oral Narrative Comprehension Improvement of Oral Narrative Production Reading Comprehension	Recounting the story, preserving its sequence and key elements, supported by verbal mediation and visual aids like pictures and miniature objects, tools that enhanced recall and helped organize coherent narratives.
Phonological Awareness	Enhancement of metaphonology of the following levels: rhyme, syllable, phoneme Reading accuracy	Large-format board games were adapted to include phonological awareness tasks, such as the identification of word count in spoken sentences, word and syllable segmentation, rhyming, syllable and phoneme identification (in various positions), and advanced phonemic operations like blending, manipulation, and transposition.
Rapid naming	Support of visual word recognition. Visual Input Formation of mental representation. Expansion of the orthographic lexicon	Large-format RAN boards were gradually introduced, progressing from illustrated to written words and pseudowords. Activities involved choral and individual naming, with varied prompting strategies to sustain engagement.
Phonological Working Memory	Promotion of auditory memory. Strengthening of the ability to follow multi-step information. Reading comprehension	Listening to sequences of words and repeating them in both original and reverse order, including sequences of syllables from target words. Additionally, a group memory game involved children sequentially recalling and adding words from the book.
Visual input	Strengthening of metaphonology Improvement of visual lexicon. Promotion of automatic recognition of high-frequency words to reduce working memory load.	Reading of Isolated Words: each child received a word card to read, followed by phonological awareness tasks (e.g., syllabic and phonemic segmentation, alliteration, and rhyme recognition). Children were then instructed to locate the word (e.g., pato) within the story text and highlight it. They also searched for other words with similar syllabic structures.
Orthographic awareness	Memory consolidation and sight word recognition, particularly via handwriting Metalinguistic skills Reflection and self-monitoring *From the third book onwards	Writing of Isolated Words: spelling key words on individual flashcards during dictation activities. Then a crossword puzzle using the target vocabulary was completed, and a table was filled out to document features of each target word (identification of the number of syllables, letters, and phonemes).
Grammar and syntactic awareness	Comprehension, by supporting idea organization and meaning construction. Reading fluency, as a result of strengthened lexical retrieval and automaticity *From the third book onwards	Sentence completion using visual prompts and color-coded structures to distinguish subjects and predicates. Later, individual words were placed on colored cards to represent grammatical classes (red for nouns, green for verbs, blue for adjectives). Colors were eventually also used to indicate sentence position—beginning, middle, and end—to support organization and comprehension.

*At-home Component: All activities were introduced during the session to guide home-based practice throughout the week. Each child received a worksheet featuring ten target words from the book. With family support, they were instructed to (1) copy each word, (2) segment it into syllables, (3) generate a new word beginning with the same syllable, (4) find a rhyming word, and (5) count the number of letters and phonemes. These activities were designed to reinforce metaphonological skills on days without intervention. Families were advised to complete the activities using two words per day, ensuring consistent and distributed reflection on language units—rhyme, syllables, and phonemes—across multiple complexity levels. A table recording the number of syllables, letters, and phonemes for each target word was also to be completed.

### 2.4 Statistical analysis

Age, years of schooling, and IQ were compared across the three diagnostic groups (ADHD, BIF and arDYS) enrolled in LIRTI^2^ using Kruskal–Wallis tests, given departures from normality on Shapiro–Wilk checks. When the omnibus test was significant, Dunn's pairwise tests with Holm correction were applied. To increase robustness to non-normality and unequal group sizes and to provide interval estimates, bias-corrected and accelerated 95% confidence intervals (BCa 95% CIs) via bootstrap resampling (1,000 samples) for pairwise differences were obtained. Sex distribution across groups was tested with chi-square tests of independence.

To evaluate post-intervention differences in reading speed, an analysis of covariance (ANCOVA) was employed with post-intervention reading speed as the dependent variable, diagnosis (three levels; ADHD, BIF and arDYS) as the fixed factor, and baseline reading speed, and school year, as covariates. Assumptions were checked as follows: homogeneity of variances, with Levene's test, and normality of residuals, with Shapiro–Wilk and Q–Q plot visual analysis. When the diagnosis effect was significant, we compared estimated marginal means (EMMs) across groups using Tukey-adjusted pairwise contrasts; BCa 95% CIs based on 1,000 bootstrap samples were reported alongside adjusted (p) values.

Reading comprehension (ordinal: Low, Medium, High) was examined using 3 × 3 chi-square tests of independence at pre-test and post-test. Cramér's (V) was reported as effect size and adjusted standardized residuals were inspected to identify cells driving the significant association.

## 3 Results

### 3.1 Group comparisons

 Table 4 presents the characteristics of the sample (sex, age, grade level, and IQ) stratified by diagnostic group: Attention-Deficit/Hyperactivity Disorder (ADHD), Borderline Intellectual Functioning (BIF) and at risk for Dyslexia (arDYS). The assessment of intellectual functioning was conducted using the Wechsler Intelligence Scale for Children, Third and Fourth Editions (WISC-III and WISC-IV (62, 63), with the Full Scale Intelligence Quotient (FSIQ) derived from the ten core subtests serving as the composite score. Even when derived from different versions of the Wechsler Intelligence Scale for Children (WISC-III or WISC-IV), IQ values remain valid and reliable indicators of general intellectual functioning. Therefore, IQ scores were considered an appropriate criterion for participant inclusion in the present study. It is important to clarify that both the WISC-III and WISC-IV provide valid and reliable measures of full-scale IQ. The core construct of general intellectual ability assessed by these instruments remains consistent across versions. Therefore, the IQ values obtained from either edition are considered a valid and useful measure for applying the study's inclusion criteria, ensuring the consistency of our participant group in terms of cognitive ability.

**Table 4:** Sample demographic characteristics by diagnostic group.

Descriptive characteristics	Diagnosis	*N*	Mean	Standard Deviation
School Year	ADHD	39: 16 girls; 23 boys	3.49	1.41
	BIF	14: 6 girls; 8 boys	2.93	1.07
	arDYS	37: 17 girls; 20 boys	3.24	1.50
Age	ADHD	39: 16 girls; 23 boys	9.05	1.61
	BIF	14: 6 girls; 8 boys	8.64	1.28
	arDYS	37: 17 girls; 20 boys	8.81	1.61
IQ	ADHD	39: 16 girls; 23 boys	102.72	10.70
	BIF	14: 6 girls; 8 boys	83.71	7.74
	arDYS	37: 17 girls; 20 boys	105.76	12.47

*N*, number of participants; ADHD, attention-deficit/hyperactivity disorder; BIF, borderline intellectual functioning; arDYS, at risk for dyslexia; IQ, intelligence quotient.

As expected, groups differed only in IQ [Kruskal–Wallis H(2) = 30.23, *p* < .001; *η*2_rank = 0.325]. Participants with BIF had lower IQ (mean = 83.71, SD = 7.74) than those with ADHD (mean = 102.72, SD = 10.70; Dunn–Holm z = 4.692, *p* < .001, rrb = 0.905) and arDYS (mean = 105.76, SD = 12.47; z = 5.386, *p* < .001, rrb = 0.921); ADHD vs. arDYS did not differ (z = −0.994, *p* = .320, rrb = 0.154). Furthermore, there were no group differences in age [Kruskal–Wallis H(2) = 0.350, *p* = .84; h2 = 0.000]; school year [H(2) = 1.476, *p* = .48; h2 = 0.000]; or association between diagnosis group and sex [c2(2) = 0.189, *p* = 0.91].

### 3.2 Reading speed across groups

Reading speed, measured in words per minute from orally read texts according to the students' grade level, is presented in Table 5, which shows reading speed across groups pre- and post-intervention.

**Table 5:** Reading speed across groups pre and post intervention.

Diagnosis	*N*	Pre-intervention		Post-intervention	
		Mean	Standard Deviation	Mean	Standard Deviation
ADHD	39	56.9	29.7	79.8	27.8
BIF	14	35.5	23.6	50.2	25.4
arDYS	37	37.0	24.9	55.1	24.3

*N*, number of participants; ADHD, attention-deficit/hyperactivity disorder; BIF, borderline intellectual functioning; arDYS, at risk for dyslexia.

An analysis of covariance (ANCOVA) was conducted to test whether post-intervention reading speed differed among the three diagnostic groups (ADHD, BIF, arDYS). Model diagnostics supported ANCOVA assumptions (residual normality: Shapiro–Wilk (W = 0.974, *p* = .070); homogeneity of variances: Levene's [F(2,87) = 1.74], (*p* = .181)). The diagnosis effect was significant after adjusting for baseline reading speed and school year [F(2,85) = 4.345, *p* = 0.016; partial *ω*^2^ = 0.069]. Baseline reading speed showed a large effect [F(1,85) = 105.075, *p* < 0.001; partial *ω*^2^ = 0.536], whereas school year did not [F(1,85) = 0.009, *p* = 0.924; partial *ω*^2^ = 0.000]. Bootstrapped *post-hoc* comparisons for Diagnosis (Tukey-adjusted *p*-values; BCa 95% CIs, 1,000 bootstraps) indicated that ADHD had higher adjusted post-intervention reading speed than BIF (*p* = 0.034) and arDYS (*p* = 0.047). The comparison between BIF and arDYS was not significant (*p* = 0.742) (Table 6).

**Table 6:** Pairwise comparisons of adjusted post-intervention Reading speed (EMM contrasts) across diagnostic groups.

Groups		Mean Difference (wpm)	95% bca CI		SE	pTukey
			Lower	Upper		
ADHD	BIF	12.936*	4.953	23.562	4.697	.034
	arDYS	9.449*	1.813	18.212	4.189	.047
BIF	arDYS	−3.652	−12.463	3.832	3.985	.742

Values are mean differences (in words per minute), with bias-corrected and accelerated 95% confidence intervals (BCa 95% CI) based on 1,000 bootstrap resamples, standard errors, and Tukey-adjusted *p*-values.

ADHD, attention-deficit/hyperactivity disorder; BIF, borderline intellectual functioning; arDYS, at risk for dyslexia; EMM, estimated marginal mean; SE, standard error; wpm, words per minute; pTukey, Tukey-adjusted *p*-value.

* *p* < 0.05.

### 3.3 Reading comprehension categories distribution across groups

**Figure 2:**
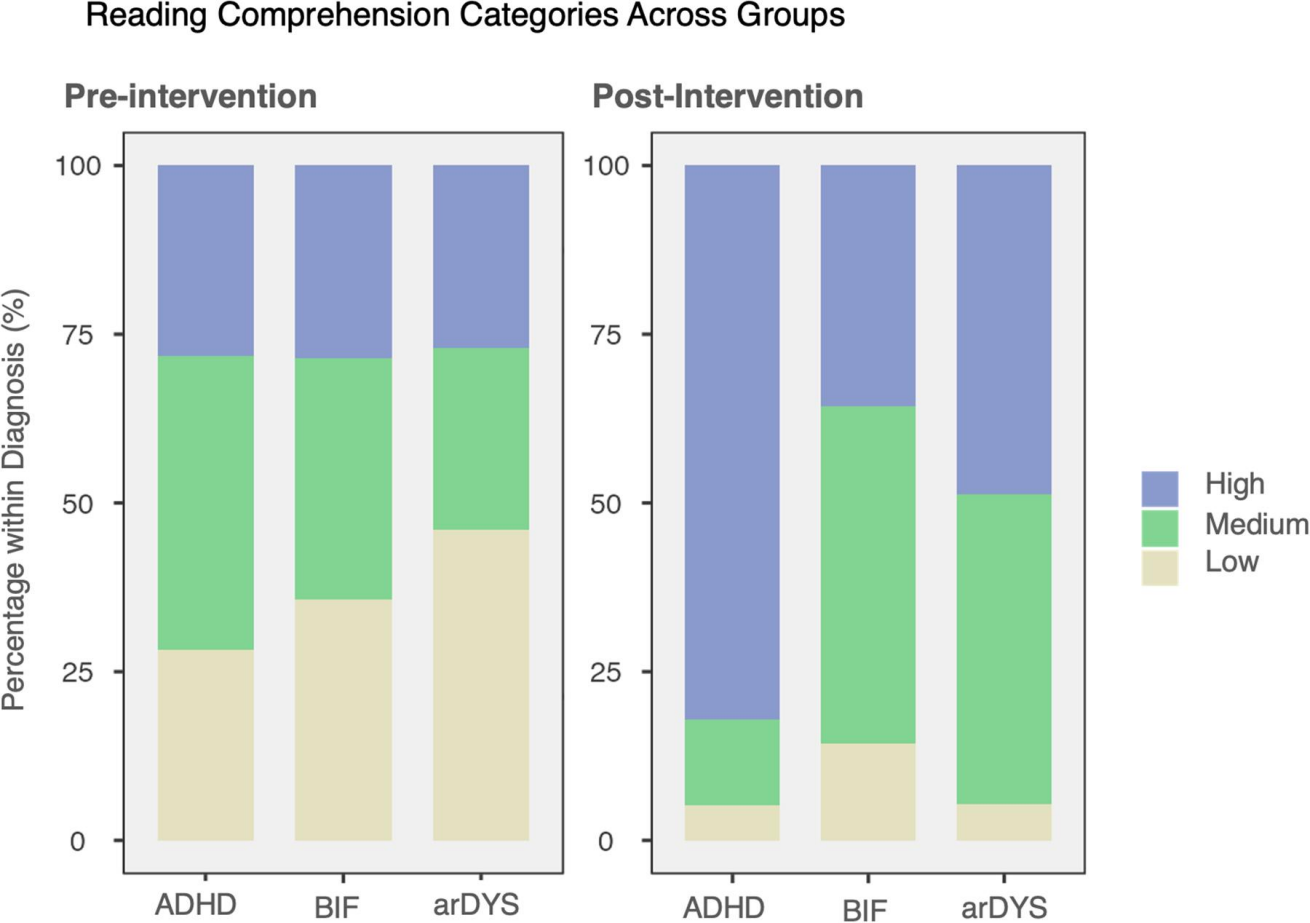
Distribution of reading comprehension categories by diagnostic group (pre- and post-intervention). Panels display the percentage of participants within each diagnostic group (ADHD, BIF, arDYS) classified as High, Medium, or Low comprehension (within-group percentages sum to 100%). Pre-intervention. Post-intervention. ADHD, attention-deficit/hyperactivity disorder; BIF, borderline intellectual functioning; arDYS, at risk for dyslexia.

At pre-test, there was no association between diagnosis (ADHD, BIF and arDYS) and comprehension level [low, medium and high; 3 × 3; *N* = 90; c^2^(4) = 3.112, *p* = 0.539]; The distribution of comprehension profiles was similar across groups (e.g., “High”: ADHD 28.2%, BIF 28.6%, arDYS 27.0%; Figure 2/Pre).

After LIRTI^2^, nevertheless, the association was significant [3 × 3; *N* = 90; c^2^(4) = 14.75, *p* = 0.005] (Cramer’s V = 0.29/Figure 2/Post). Adjusted standardized residuals showed that in ADHD, “High” comprehension was higher than expected [32/39 = 82.1%; (r = +3.56)], “Medium” lower than expected [5/39 = 12.8%; (r = −3.44)], and “Low” was rare [2/39 = 5.1%; (r = −0.51)]. In contrast in BIF, “High” was lower than expected [5/14 = 35.7%; (r = −2.12)] and “Medium” tended to be higher [7/14 = 50.0%; (r = +1.55)]; and “Low” remained infrequent [2/14 = 14.3%; (r = +1.24)]. Finally, for the arDYS group, “High” was again lower than expected [18/37 = 48.6%; (r = −2.03)] and “Medium” higher [17/37 = 45.9%; (r = +2.33)], while “Low” lasted uncommon [2/37 = 5.4%; (r = −0.40)]. Taken together, these results indicate that, post-LIRTI^2^, more ADHD participants reached “High” comprehension, whereas the BIF and arDYS groups showed shifted distributions toward “Medium”, explaining the significant diagnosis–comprehension association.

## 4 Discussion

By analyzing routinely collected service data, we address a critical evidence gap on Tier 2, community-based supports for at-risk readers, viable in Brazil and other contexts that limit RTI execution at the School, and patient and familiar availability to attend to frequent intervention sessions.

Diagnosis was found to significantly influence post-intervention reading fluency, after controlling for reading velocity before the beginning of the intervention, and school year. A higher post-LIRTI^2^ reading speed was observed in the ADHD children compared to both the BIF and arDYS groups. Regarding reading comprehension, before the LIRTI^2^, profiles were similar across groups. Following the intervention, nevertheless, the diagnostic group was significantly associated with reading comprehension level.

### 4.1 Reading speed by diagnostic profile

Students with ADHD showed greater reading speed compared to those with BIF or arDYS after LIRTI^2^. However, no meaningful difference was found between the BIF and arDYS groups. Other studies implementing Tier 2 of the RTI model, typically conducted more frequently and within school settings, have reported similar results. Children with ADHD benefited the most from the structured intervention, supporting prior findings that, despite challenges related to attention regulation, executive functioning (19, 20), processing speed, and working memory (67, 68), this population can respond effectively to targeted, skill-specific reading instruction. The interactive and scaffolded nature of the LIRTI^2^ model may have further enhanced engagement and information retention. Reading interventions, whether delivered alone or in conjunction with ADHD treatment, yield superior outcomes compared to ADHD treatment alone, reinforcing the benefits of reading-specific support (66).

One recent study also found that at least 30 h of phonological awareness instruction constitutes an evidence-based intervention for the improvement of reading for this group (69). The present findings are clinically relevant given evidence that individuals diagnosed ADHD in childhood continue to show deficits in reading speed and comprehension into adulthood (70). Notably, fluency difficulties were observed even among participants with otherwise adequate basic reading skills, as word recognition. In this context, the proposed LIRTI^2^ may play an important role by systematically targeting fluency while supporting comprehension, offering a viable and scalable option for learners who do not require Tier 3 services yet fail to progress with core classroom instruction.

In contrast, students with BIF and arDYS showed more constrained reading velocity after LIRTI^2^ although for differing reasons (71). Similar results were found in research in RTI 2 tier, which indicate that tier 3 interventions within the RTI framework are typically necessary for students with more persistent and severe learning difficulties, such as those associated with Dyslexia and BIF. These students often require individualized, intensive instruction that goes beyond the support provided in Tier 2 (72).

The BIF group's limited velocity is consistent with previous findings that attribute intervention resistance to broad cognitive inefficiencies, including reduced working memory, processing speed, and learning consolidation (71, 73).

Although some children with BIF may achieve decoding accuracy when given sufficient time, their slowness in reading imposes a heavy cognitive load, particularly on phonological working memory, limiting their ability to process and integrate textual information efficiently (74). They often remain significantly below normative expectations, as evidenced in longitudinal and case study data (75). This suggests that their difficulties with fluency are not solely rooted in reading-specific deficits, but are exacerbated by global cognitive limitations, such as inefficient information processing and limited executive functioning (73).

Children arDYS also demonstrated modest rates in reading speed, which aligns with a well-established body of literature identifying fluency as a core, persistent deficit in this population (76). Dyslexia has been increasingly framed as a disorder marked by durable challenges in automatic word recognition and phonological processing, regardless of the language's orthography system (16). Even when systematic phonics-based instruction is provided, as in the Brazilian RTI study (45), fluency deficits often remain.

This phenomenon is linked to multiple neurocognitive factors that hinder the development of automatic reading, thereby impairing fluency (77, 78). One of the primary contributors to this persistent slowness is the phonological deficit, which may impair the ability to manipulate the sounds of language and results in inefficient decoding (76). Consequently, dyslexic readers often rely heavily on laborious, conscious decoding strategies, which significantly slow down their reading speed (78).

Additionally, poor integration between phonological and visual processing systems may contribute to the delayed automatization of grapheme–phoneme correspondences (79). Moreover, individuals with Dyslexia typically struggle to form stable and detailed orthographic representations, which are essential for the automatic recognition of words (80). This difficulty interferes with the transition from phonological decoding to fluent word recognition, a critical step in becoming a proficient reader.

### 4.2 Reading comprehension across diagnostic profiles

The three clinical groups presented relatively similar baseline levels of comprehension on the LIRTI^2^ prior to the intervention, generally ranging from low to medium scores. After intervention, an statistical significant association was found between diagnosis groups and comprehension levels. More children with ADHD achieved the “High” comprehension category then expected. This can be explained by the nature of ADHD-related difficulties, which often involve attentional regulation rather than core linguistic impairments. Once decoding becomes automatic, children with ADHD can better allocate their limited attentional resources to comprehend (81). These findings reinforce that structured reading intervention provides significant benefits for this group.

Children with Borderline Intellectual Functioning (BIF) showed more modest comprehension levels, which can be at least partly accounted by broader cognitive challenges in this population—namely deficits in working memory, processing speed, and executive function (78, 79).

Furthermore, these students typically present with additional language-related challenges, including restricted vocabulary, reduced morphosyntactic awareness, and narrative difficulties, that further constrain their ability to compensate for slow reading speed (82). As such, their fluency difficulties contribute to weaker comprehension outcomes, as they struggle to maintain coherence across sentences and retain information over longer passages. For these students, nevertheless, fluency-focused interventions may be insufficient on their own, highlighting the need for more comprehensive approaches that integrate explicit language instruction, vocabulary development, and comprehension strategies (82, 83).

After LIRTI^2^, children arDYS achieved “Medium” comprehension more often than expected. This underscores their potential to mitigate some of the challenges posed by the deficit (83). Even though dyslexic children read at a slower pace, a modest increase in reading speed may yield meaningful benefits for their comprehension, as it can reduce the cognitive load on phonological working memory (79). This, in turn, enhances their ability to retain and integrate information across the text, especially as they develop compensatory strategies to support comprehension (84). While dyslexic readers often struggle to transition from phonological decoding to automatic word recognition due to underdeveloped orthographic representation (73), strategies such as repeated reading, assisted reading, and prosody training can enhance fluency and free up cognitive resources for comprehension (85).

Collectively, these findings highlight the essential role of reading speed in supporting reading comprehension (86, 87), although speed alone is not sufficient. Effective comprehension depends on the integration of multiple cognitive and linguistic components, including vocabulary knowledge, syntactic processing, background knowledge, and executive functioning (88–90). Therefore, interventions must extend beyond reading speed to target these foundational elements by adopting a comprehensive approach that incorporates higher-order skills such as vocabulary development, explicit instruction in comprehension strategies, and motivational support to address the multifaceted nature of reading difficulties (25, 26).

### 4.3 Adapting tier 2 interventions to structural realities

Interventions grounded in multi-tiered systems of support for children with reading difficulties have shown consistently positive results. Particularly, Tier 2 of the Response to Intervention (RTI) framework is characterized by small-group, focused instruction delivered intensively (three to five times per week) has proven to significant improvements in reading fluency and academic outcomes for students with reading difficulties. Research has demonstrated significant gains in reading fluency as a result of such interventions (41, 91, 92). However, implementing supplemental reading programs for struggling readers remains particularly challenging in low-resource contexts, where barriers often limit access and continuity (93).

Embedding Tier 2 interventions within school settings, as originally conceptualized, may improve accessibility for vulnerable student populations. However, in Brazil, structural challenges, such as part-time school schedules, overcrowded classrooms, and lack of specialized teachers, significantly hinder participation in supplementary educational programs (94).

There are other factors such as vulnerability, and lack of access to transportation or parental availability that often prevent children from participating. These challenges are especially pronounced in densely populated urban areas like Rio de Janeiro, were inequities in support limit access for at-risk learners. The present study's age profile (mean 9 years old) aligns with prior reports of systematic delays in diagnostic assessment and initiation of appropriate intervention, suggesting that many children access services later than clinically advisable (95). These contextual barriers, spanning both educational provision and the healthcare system, likely exacerbate reading difficulties and impede early identification and intervention (96).

There is little doubt that children would benefit more, or benefit more quickly, from intensive intervention programs; however, the challenges of implementing such practices are considerable. These considerations underscore the importance of aligning intervention models with the structural and contextual realities in which they are applied. RTI Tier 2 interventions for children with reading difficulties can be adapted to account for structural inequalities, ensuring not only high instructional quality and fidelity but also logistical viability within under-resourced scenarios (97, 98).

To address these issues, an adaptation approach, LIRTI^2^ (Less Intensive Response to Intervention—Tier 2) was structured. One of its key adaptations is that it is delivered outside the traditional school setting. Another important distinction is its lower weekly frequency: sessions were held only once per week, but over a longer duration than typical programs—a total of 18 weeks, with each session lasting two hours. Despite these adaptations, the program maintains core elements of Tier 2 RTI approaches. It provides supplemental small-group instruction (27, 99), and its instructional methodology is grounded in systematic and explicit teaching practices (50, 100), focusing on the development of phonological decoding, word recognition, and oral reading fluency (24).

The delivery of Tier 2 interventions in specialized clinical contexts constitutes a principled adaptation of the Response to Intervention (RTI) framework. We argue that fidelity to the core logic of Tier 2 is maintained through: (1) providing standardized, evidence-based intervention to a targeted group; (2) using frequent progress monitoring to inform instructional adaptation; and (3) establishing clear decision rules for judging non-response. By shifting the setting from school to clinic, this model offers a structured, diagnostic intervention trial characterized by controlled conditions and expert implementation for students demonstrating insufficient response to initial school-based support. This clinical-tier approach is theoretically grounded in integrated service models, such as the Interconnected Systems Framework (ISF), which formally embeds specialists within multi-tiered support systems to address complex student needs (101). Empirically, it aligns with evidence demonstrating that standardized, small-group interventions, when delivered with high fidelity, can effectively remediate skill deficits for a substantial proportion of at-risk learners (99, 102). Consequently, the clinical setting functions not as a separate, alternative system, but as a specialized extension of the school's (Multi-Tiered System of Supports (MTSS) continuum, providing an intermediate level of support prior to consideration for maximally individualized (Tier 3) services. Thus, while the context and dosage schedule differ from traditional school-based Tier 2, the model maintains comparability in its functional components and decision-making logic, allowing for possible comparison.

Accordingly, LIRTI^2^ operationalizes a Tier 2, equity-oriented adaptation: it reconfigures dose and setting to fit the structural realities that constrain access, without abandoning core RTI principles like explicit, skills-based instruction and supplemental small-group work. Policy-wise, aligning RTI-inspired supports with Brazil's framework could enhance early identification and timely Tier 2 access. It is important to emphasize that this does not replace the need for the first tier of RTI, that is, the universal implementation of evidence-based literacy development. On the contrary, implementing Tier 2 of RTI makes much more sense—and would likely involve fewer children—if Tier 1 were fully ensured in regular classroom instruction.

### 4.4 Bridging educational interventions and health

Despite the adoption of RTI frameworks, tier 2 interventions have been the subject of critical debate. One common critique concerns the use of a single, uniform intervention model to address a wide range of reading difficulties (103). Nonetheless, given that the foundational components of reading, as phonemic awareness, decoding, fluency, and comprehension, are well-established predictors of reading success, providing targeted support in these domains is warranted to optimize fluency gains (104). This does not preclude adding or adapting interventions in other domains or at a later stage, if required.

Another concern relates to the structure of the RTI model itself, where students are often required to “fail” Tier 2 supports before access to more intensive Tier 3 interventions. The concern is that this reactive progression may delay timely identification of students with significant learning needs, potentially hindering early and effective support (103, 105). However, in the Brazilian context, this particular criticism of RTI does not appear to be supported by empirical evidence since one notable characteristic of the Brazilian context is the late diagnosis of learning difficulties, as previously discussed (95). As the intervention is often not embedded in the school setting, families typically access specialized services only at later stages.

Therefore, adaptations of the RTI (Response to Intervention) model, like LIRTI^2^ that are viable in different contexts can help children with persistent reading difficulties to be referred to healthcare services much earlier. This premise aligns with a Delphi study (17), which emphasizes the urgent need for early support for all children showing difficulties at the onset of reading, and underscores that effective intervention must be rooted in the principle of equitable access to instruction.

Research consistently shows that individuals struggling with reading, regardless of socioeconomic background or diagnostic risk, require timely, targeted support, including continuous progress monitoring and access to instructional resources. Delaying assistance can impede learning and aggravate existing achievement gaps (95, 106). Early intervention should be based on observable performance indicators, with more comprehensive assessments introduced as needed (16).

Furthermore, the healthcare system can also potentially benefit from the dissemination of initiatives like LIRTI^2^, that can help to ensure that only those truly in need of specialized medical evaluation are referred to health professionals. This targeted approach not only may optimize the use of limited resources but also eases pressure on already overstretched services. Providing timely, tailored educational support to children at risk for neurodevelopmental disorders, but who do not require clinical intervention, may reduce unnecessary referrals and could allow specialized care to be focused on those with more complex needs (107).

Furthermore, scaling initiatives such as LIRTI^2^ may optimize healthcare resource use by restricting referrals for specialized assessment to cases with clear clinical indication.

The integration of LIRTI^2^ approaches in after-school or community settings has the potential to foster early literacy by broadening access to reading support beyond traditional classrooms (108), offering a low-cost scalable complement to core instruction for underserved populations.

### 4.5 Conclusion, limitation and future directions

This retrospective, service-based study describes LIRTI^2^, a Tier 2 intervention within the RTI framework, and reports a diagnostic effect on post-intervention reading speed, and comprehension, with Attention-Deficit/Hyperactivity Disorder (ADHD) outperforming and Borderline Intellectual Functioning (BIF) and at risk for Dyslexia (arDYS) children. Several limitations warrant caution. First, the observational design without a concurrent control group precludes causal inference and leaves open the possibility of unmeasured confounding, and selection bias. Second, the BIF subgroup was relatively small, limiting precision and generalizability; findings should be replicated in larger, more diverse samples and, where feasible, in multisite implementations. Third, reading comprehension was measured with a small number of literal items per passage and summarized in broad percentage bands (low/medium/high), likely reducing metric sensitivity, and masking finer gains in inferential or integrative comprehension. Finally, longitudinal studies with follow up are needed to assess the sustainability of gains and their broader academic impact.

Notwithstanding these limitations, LIRTI^2^-like Tier 2 adaptations can expand access to fluency-focused, explicit instruction in under-resourced settings. By triaging non-responders to more specialized care, these programs may streamline the healthcare system–level referral pathway. Prospective evaluations should confirm which learner profiles benefit most to guide targeted scale-up.

## Data Availability

The datasets presented in this article are not readily available because all data were handled in accordance with ethical guidelines and are protected to ensure participant confidentiality. Requests to access the datasets should be directed to the corresponding authors.
